# Exploration and augmentation of pharmacological space via adversarial auto-encoder model for facilitating kinase-centric drug development

**DOI:** 10.1186/s13321-021-00574-4

**Published:** 2021-12-06

**Authors:** Xinyu Bai, Yuxin Yin

**Affiliations:** 1 Department of Pathology, School of Basic Medical Sciences, Peking University Health Science Center, Beijing, 100191 China; 2 Institute of Systems Biomedicine, School of Basic Medical Sciences, Peking University Health Science Center, Beijing, 100191 People’s Republic of China; 3 Peking-Tsinghua Center for Life Sciences, Peking University Health Science Center, Beijing, 100191 China

**Keywords:** Data augmentation, AAE, Cheminformatics, Deep learning, Kinase

## Abstract

Predicting compound–protein interactions (CPIs) is of great importance for drug discovery and repositioning, yet still challenging mainly due to the sparse nature of CPI matrixes, resulting in poor generalization performance. Hence, unlike typical CPI prediction models focused on representation learning or model selection, we propose a deep neural network-based strategy, PCM-AAE, that re-explores and augments the pharmacological space of kinase inhibitors by introducing the adversarial auto-encoder model (AAE) to improve the generalization of the prediction model. To complete the data space, we constructed Ensemble of PCM-AAE (EPA), an ensemble model that quickly and accurately yields quantitative predictions of binding affinity between any human kinase and inhibitor. In rigorous internal validation, EPA showed excellent performance, consistently outperforming the model trained with the imbalanced set, especially for targets with relatively fewer training data points. Improved prediction accuracy of EPA for external datasets enhances its generalization ability, making it possible to gracefully handle previously unseen kinases and inhibitors. EPA showed promising potential when directly applied to virtual screening and off-target prediction, exhibiting its practicality in hit prediction. Our strategy is expected to facilitate kinase-centric drug development, as well as to solve more challenging prediction problems with insufficient data points.

**Supplementary Information:**

The online version contains supplementary material available at 10.1186/s13321-021-00574-4.

## Introduction

Determining the compound–protein interactions (CPI) is a key aspect in drug development, contributing to both understanding complicated mechanism of action (MoA) of drugs and discovering novel inhibitors of proteins [1]. In particular, for the homologous proteins with high genetic conservation and similar structures (e.g. protein kinase families), it is necessary and challenging to detect the protein-inhibitor profiling which may bring therapeutic effects or side effects. However, the number of activity matrixes is still sparse due to the time-consuming and cost-intensive efforts for establishing and conducting biological assays [2]. To increase the efficiency of this process, various structure- and ligand-based computational approaches have been developed for pre-screening [3, 4], yet their predictive power are limited in many cases partly because their accuracy depends heavily on a decent number of active compounds towards the targets [5].

Recently, deep learning (DL) has achieved remarkable predictive power by extrapolating the pattern from large amounts of data in a diverse range of applications such as natural language processing, speech recognition, and computer vision [6]. Encouraged by such success and rapid growth of biomedical data, increasing interest in applying DL methods to accelerating drug development has spawned many efforts in this field, such as protein structure prediction, retrosynthetic planning, and de novo drug design with desirable properties [7–9]. With the accumulation of CPI-related databases, DL techniques have also been recruited to improve the accuracy of CPI prediction. Although the CPI problem has been considered as a binary classification task in a number of studies, such as DeepDTI and DeepConv-DTI and GraphDTI [10–12], given that binding affinity represents CPI strength, many studies have been devoted to developing regression models to predict binding affinity, for instance, DeepDTA uses a convolutional neural network (CNN) fed with the protein sequences and compound SMILEs to extract CPI patterns [13], while GraphDTA represents compounds as graphs to train Graph CNN (GCN) models [14]. Such methods mainly focus on novel algorithm establishment and representation exploration, nevertheless, even though experimental uncertainty and the sparseness of CPI annotations set limits for DL models greatly [15], studies on improving the quality of training data are few.

One natural way to overcome the aforementioned problems is data augmentation for CPI annotations. Specifically, the binding affinity prediction task with a lack of activity annotations can be considered as an imbalanced regression problem, where the data have imbalanced distributions and certain target values were fewer observed, hindering the predictive performance of the regression models [16]. The approaches addressing this problem mainly include resampling continuous observations for data balancing (e.g. SmoteR) and proposing evaluation metrics that take varying importance of observations into consideration [17, 18]. A recent study proposed another straightforward strategy to rise to the challenge, which labels additional training data points using established prediction models, then trains a standard prediction model with augmented datasets [19]. All these studies emphasize data that are more balanced would enhance the generalization ability of the CPI model.

Recent studies on data augmentation based on generative models have led to a clearer margin among different data categories [20–22], inspiring us to utilize the generative models for tackling the imbalanced regression problem of CPI by generating samples with activity annotations. Thus, we proposed two strategies, PCM-GAN and PCM-AAE, to expand the data space of the protein kinase-inhibitor datasets, which was then employed for predictor construction to predict kinase protein-inhibitor interaction. To further improve the predictive accuracy, we proposed Ensemble of PCM-AAE (EPA) to integrate the data space by random forest (RF), and demonstrated its superiority by evaluation with internal and external datasets.

## Methods

### Data collection, preprocessing and analysis

Six kinase bioactivity datasets were collected for generating and validating the prediction models: Christmann-Franck’s dataset [23], Kinase SARfari [24], PKIS1 [25], MRC (MRC PPU, https://www.ppu.mrc.ac.uk/), Metz’s dataset [26] and Davis’s dataset [27]. The numbers of molecules and kinases in the datasets are summarized in Table 1. The proteins in the datasets were grouped by their Uniprot Identifier, while compounds were integrated based on their canonical SMILEs. The sequence of the kinase domain was extracted from the full sequence of each kinase.

**Table 1:** Statistics of datasets

Datasets	Compounds	Kinases	Data points
Christmann-Franck’s	2000	196	85,958
Kinase SARfari	9977	20	20,508
PKIS1	354	187	66,197
MRC	217	116	27,154
Metz’s	1450	161	99,480
Davis’s	70	336	6642

In previous work, Christmann-Franck et al. have presented the activity standardization protocol and developed proteochemometric models for activity predictionn. We followed their work and, based on which we trained models with Christmann-Franck’s dataset and validated their performance on the other 5 datasets. Given that the latter 5 datasets are heterogeneous and obtained by various biological methods, we referred to the activity standardization protocol proposed by Christmann-Franck et al. to preprocess the data: (1) the data with units of pKi, pKd, Kd, Ki, and POC (Percent of Control) were selected to ensure the data employed for model building represent the compound activity against kinases rather than the cell lines; (2) then the selected data were standardized with rules reported previously [23]. Specifically, the concentration units of all measures were unified to nanomolar, where Ki, Kd and POC values were then − log10 transformed to pKi/pKd; (3) to remove duplicates, the coefficient of variation (CV) of repeated measurements were calculated for each dataset. If the CV value for repeated measurements is over 0.05, then the measurements were removed, otherwise one of the repeated measurements was kept randomly. The cutoff to distinguish active compounds from inactive ones was set at 6 (corresponding to the measured value of 1 µM, a commonly used threshold).

### Data representations

Vector embeddings of compounds were obtained through Mol2Vec model [28]. The compound corpus was obtained from ZINC database version 15 [29], where the canonical SMILES representation of each compound was transformed to a list of ordered atom identifiers as a “molecule sentence” through Morgan algorithm. To embed the “molecule sentence” of a specific molecule, each atom identifier was converted to a 100- or 300-dimensional vector by skip-gram, and every molecule was represented as the vector sum of its atom identifiers.

Vector embeddings for proteins were produced with ProtVec model [30]. The protein corpus of 554,241 sequences was downloaded from Swiss-Prot [31]. Each protein sequence was represented as three 3-g sequences. A total of 1,662,723 (554,241 × 3) sequences were generated for Protvec model training, where each 3-amino acid phrase was converted to a 100 or 300-dimensional vector. For each protein-compound pair, we explored three different combination methods of compound and protein sequence embeddings (Additional file 1: Fig. S1a). As the results shown in Additional file 1: Fig. S1b, Combination 2 (concatenating 300-dimensional compound embeddings with the sum of three 300-dimensional 3-g protein sequence embeddings) showed slightly better performance than Combination 1 (concatenating 100-dimensional compound embeddings with the sum of three 100-dimensional 3-g protein sequence embeddings) and Combination 3 (concatenating 300-dimensional compound embeddings with the concatenation of three 100-dimensional 3-g protein sequence embeddings). Therefore Combination 2 was selected for the subsequent feature extraction of all datasets.

### Baseline model building

Random forest (RF) and deep neural network (DNN) regressors are implemented in Scikit-learn. To select the most suitable combination of parameters, grid search was performed. In the final RF model, the number of estimators was set at 500 and the maximum number of features was set at 200. In the final DNN model, three hidden layers are activated by rectified linear unit (ReLU), where each layer is comprised of 200 neurons. The output layer is activated by the sigmoid function. ADAM optimization algorithm is used for weight optimization [32].

### Architecture of PCM-GAN and PCM-AAE

**Fig. 1:**
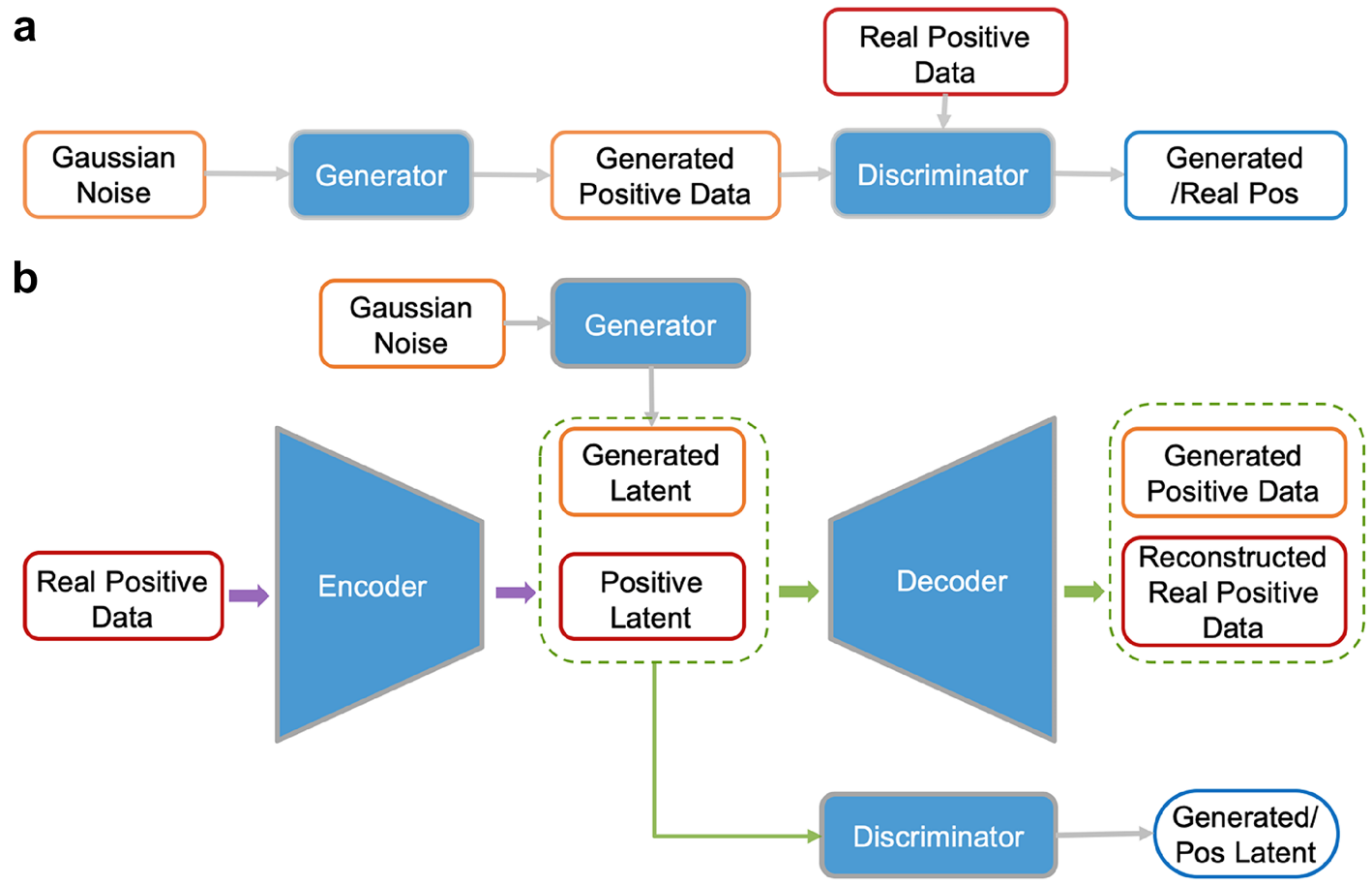
The architecture of **a** PCM-GAN and **b** PCM-AAE

Generally, GAN trains generator (G) and discriminator (D) jointly until G generates fake data that match the distributions of real data and these fake data cannot be distinguished from real data by D [33]. In PCM-GAN (Fig. 1a), noise input randomly sampled from normal distribution was fed to the generators, generating the data to fool the discriminator, which was trained to distinguish the real positive data (− log affinity ≥ 6) and the generated data. Based on auto-encoder and GAN, AAE is jointly trained by minimizing the reconstruction error of auto-encoder and the adversarial loss for matching the aggregated posterior distribution of outputs from the encoder to the stochastic prior distribution [34]. Similar to the typical AAE model, PCM-AAE architecture (Fig. 1b) employed the section of GAN to match the distribution of the latent layer vector of the auto-encoder model to generate data by generator (generated latent). The input of the encoder was the positive data in the training set, and the input of the generator was random noises with normal distribution. In the end, a specific sampled distribution was firstly converted to a positive latent vector by the generator, and the resultant vector was then fed into the decoder to output new positive PCM2vec data.

It is noteworthy that the continuous labels corresponding to the generated samples were learned by the generative model, which means that in our models, the dimension of the input training data is 601 [600-dimensional feature vector + 1-dimensional experimental measurement (binding affinity)], and the outputs of the generator of GAN or of the decoder in AAE is the concatenation of 600-dimensional new feature vector data point and 1-dimensional label (Additional file 1: Fig. S2). Obviously, whether the generated 1-dimensional label distributed within a reasonable range partially reflects whether the generative models were trained successfully for generating valid samples. Therefore, during the training process, the ratio of generated labels should distribute between 6 and 11 (corresponding to the activity of 0.01 nM to 1 µM). Pseudocode for PCM-AAE is provided in Additional file 1: Algorithm S1.

PCM-GAN and PCM-AAE were established in Tensorflow (version 1.3.0) [35]. Fully connected layers were adopted in all modules. Models were trained with Kullback–Leibler (KL) cost annealing. ADAM with a learning rate of 0.01 was used as the optimization algorithm during the training for both generator and discriminator. The epoch number was set at 50, and the batch size was set at 64.

### PCA and t-SNE algorithms for data exploration

To explore the data space, the high-dimensional embeddings mentioned above were firstly reduced to 50 dimensions using principal component analysis (PCA) [36], a traditional technique to orthogonal-linearly transform data to their low-dimensional representatives. The top 50 principal components were selected, with a cumulative explained variance percentage of 89.65% (Additional file 1: Fig. S3a). We then mapped the 50-dimensional representations to two dimension by Barnes–Hut t-distributed Stochastic Embedding (t-SNE) algorithm [37], a popular method that captures the non-linear relationship of data. The t-SNE algorithm considers that samples are Gaussian-distributed in high-dimensional space while Student t-distributed in low-dimensional space, it learns the transformed embeddings with minimum information loss, and minimizes the KL divergence of sample distributions in high and low dimensions simultaneously [38]. PCA and t-SNE were both implemented by Python Scikit-learn [39].

### Balancing methods

When comparing our strategy with existing methods for imbalanced regression problem, random undersampling was implemented by Python Scikit-learn with RandomUnderSamp (RUS) algorithm [37], while oversampling was performed using SmoteR as described previously [18].

### Model evaluation

Here, we developed proteochemometrics (PCM) model to predict compound-kinase interactions. As previously reported, PCM model is a typical pair-input method where the test set shares components with the training set and thus tend to perform much better than those that do not [40]. Hence it is necessary to distinguish test pairs based on their component-level overlap when evaluating performance. To this end, a four-level (CV1–CV4) validation strategy has been proposed and broadly used for rigorous validation on PCM model [41], which was adopted for our model evaluation. CV1 assesses the model performance on unknown compound-kinase pairs, where compounds or kinases might have already been present in the training data. CV2 tests the model performance on new kinases by excluding kinases present in the respective training data. Accordingly, CV3 evaluates the model on new compounds, and CV4 on unknown kinases and compounds.

**Fig. 2:**
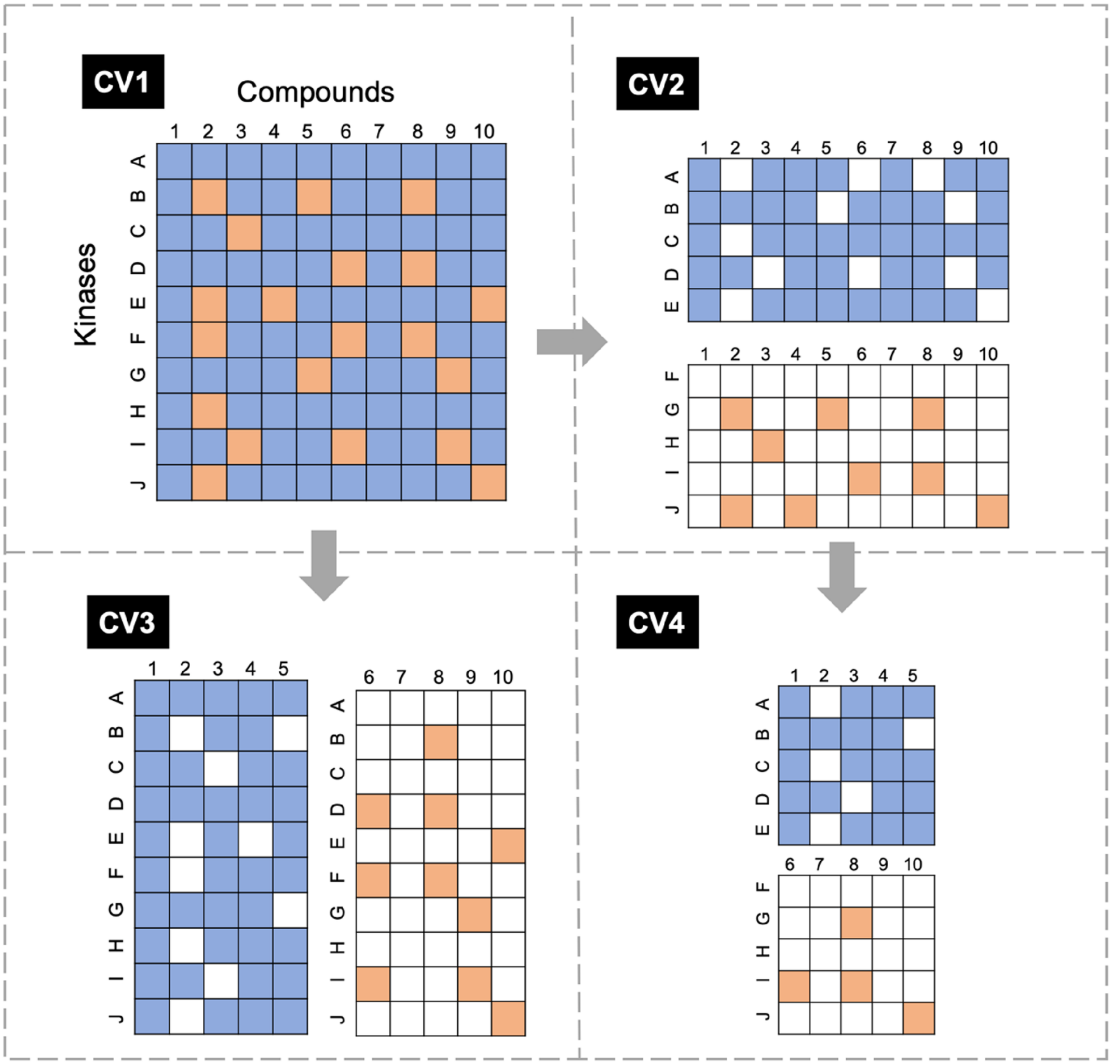
Four-level cross validation. Blue: training set, Orange: test set

The detailed method to split the dataset was illustrated in Fig. 2. In CV1, the datasets were randomly partitioned into 80% CV1 training set and 20% test set, where the numbers of negative samples and positive samples are equal. For CV2, all kinases in CV1 datasets were split into two subsets, then the CV1 training data was extracted from one subset (kinase A, B, C, D, E) as CV2 training set, and the CV1 test data was extracted from the other subset (kinase F, G, H, I, J) as CV2 test set. Similarly, for CV3, all compounds were split into two subsets (compound 1, 2, 3, 4, 5 and compound 6, 7, 8, 9, 10), where one subset (compound 1, 2, 3, 4, 5) rules out the test data as CV3 training set, and the other subset (compound 6, 7, 8, 9, 10) excludes the training data as CV3 test set. For CV4, a set of inhibitors (compound 1, 2, 3, 4, 5) in CV2 training set were extracted as CV4 training set, another set of inhibitors (compound 6, 7, 8, 9, 10) in CV2 test set were extracted as CV4 test set. It should be noted that at each CV level, the sampling of kinases or inhibitors was iterated until the ratio of training data to test data was closed to 4:1 and the numbers of positive samples and negative samples in the training set and test set were nearly equal.

As a regression problem, prediction error was measured using Pearson’s correlation coefficient (PCC), mean absolute error (MAE) and mean squared error (MSE) between experimental measurements and predicted values to assess model quality. Area under ROC (AUC), F1 score, recall score and precision score were also calculated to characterize the model capability of distinguishing different classes of the samples.

## Results and discussion

### Baseline methods comparison

To assess how effective the strategy we proposed, we first trained the baseline predictor which was fed with an imbalanced dataset (non-balanced model/NB model) using three popular machine learning algorithms: random forest (RF), XGBoost [42], and deep neural network (DNN). To speed up the convergence of DNN, each feature was standardized with zero mean and unit variance prior to feeding feature vectors. Regression accuracy of the models was evaluated by metrics of MAE and PCC, while classification accuracy was evaluated by the metrics of AUC and sensitivity. As expected, DNN models fed with standardized data (ST-DNN) achieved more rapid convergence after 30 iterations than those fed with non-standardized data (NST-DNN), with RMSE of 0.06 and 0.17 respectively. Moreover, the model performance shown in Additional file 1: Fig. S4 suggests that (1) in CV1, the ST-DNN model evidently surpasses the other methods for all metrics considered; (2) in CV3, RF and ST-DNN showed comparable performance that outperformed XGboost; (3) in CV2 and CV4, RF models demonstrated the best performance for metrics of PCC, MAE and AUC; (4) from CV1 to CV4, the F1 score of ST-DNN models were far more superior to that of other models on average, indicating the stronger capability of ST-DNN to capture positive samples. Overall, considering that the ST-DNN predictor was of the highest quality among the models evaluated in the case of the training dataset in this work, it was employed as the baseline model in further investigation. It should be noted that further fine-tuning might improve the prediction power of this baseline method.

### Implementation and training of PCM-GAN and PCM-AAE

The key assumption in this work is that the more well-portrayed the positive sample space is, the stronger the generalization ability of the predictive model. To verify this hypothesis, two popular generative models based on DNN architectures, Generative Adversarial Network (GAN) and Adversarial Auto-encoder (AAE), were introduced to generate novel PCM2vec representations, so that the positive sample space would be enlarged and alienate from the distribution of the negative samples. The resultant models were termed PCM-GAN and PCM-AAE respectively (Fig. 1).

**Fig. 3:**
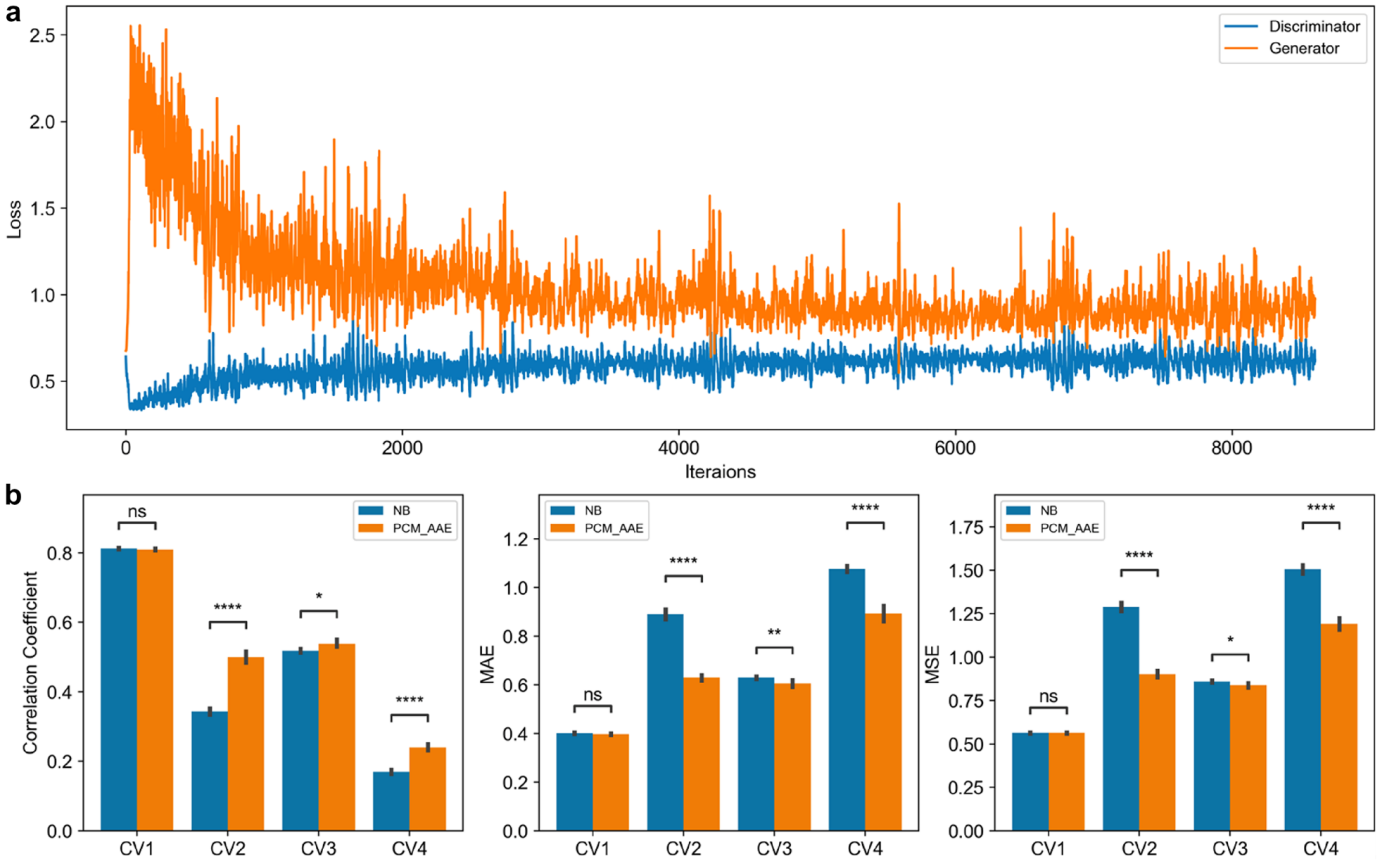
**a** Loss plot of PCM-AAE. **b** Performance comparison between non-balanced model (NB) and reconstructed model with augmented data from generators trained by PCM-AAE. Statistical significance of the difference between the performance of NB and PCM-AAE was determined by paired t-test. ns: p > 0.05; *p < 0.05; **p < 0.01; ***p < 0.001; ****p < 0.0001

As shown in Additional file 1: Fig. S5, we plotted errors of PCM-GAN models against iterations. When training PCM-GAN, we found that it is difficult for the generator and the discriminator to converge simultaneously, that is, one side was completely victorious while the other side continuously had a large error at any arbitrary point. Interestingly, PCM-AAE can achieve a win-win situation for both discriminator and generator with converged loss during the confrontation (Fig. 3a). Previous research showed that batch-normalization and dropout layer are crucial to GAN and AAE training on different datasets [34]. In our model settings, the batch-normalization and drop-out used in all layers of auto-encoder did help convergence of PCM-AAE but not PCM-GAN training. In addition, it was difficult for PCM-GAN to generate valid 1-dimensional labels, while the generator of PCM-AAE could generate more than 90% valid labels after it was trained for 1000 iterations. A potential explanation is that compared with 601-dimensional data, it is easier for PCM-AAE to capture the distribution of low-dimensional data resultant by auto-encoder; or that auto-encoder is good at learning representation that is more effective in a low dimensional space [43].

Next, we assessed whether the generated samples from PCM-AAE are useful for improving the performance of NB models trained above. PCM-AAE was firstly trained with the CV4 training set which was sampled from the CV2 training set and had no overlap with the test sets in CV1–CV4. Here, 24 generators were randomly saved. Then a certain number of generated samples from the generators were added to the training set of CV1–CV4 to train DNN predictors again, which were used to predict the test set of CV1–CV4 respectively. To compare the performance of the balanced model by PCM-AAE with non-balanced (NB) models, 20 NB models and 20 balanced models were trained at each CV level respectively (Additional file 1: Fig. S6). Overall, the models with data complemented by the generators showed more superior performance than the NB models, especially, the model obtained by the best AAE (AAE #14 in Additional file 1: Fig. S6) showed significant improvement compared with the NB model in CV2–CV4 (Fig. 3b).

### Exploration of PCM-AAE data space

To illustrate the effectiveness of PCM-AAE for data augmentation, the PCM2vec was mapped to low-dimensional space by t-SNE algorithm, so that the alteration of the data space could be demonstrated by data visualization. However, directly applying t-SNE to a large-sized training set to reduce 600-dimensional PCM2vec is rather inefficient, therefore PCA was used prior to t-SNE to accelerate this process (Additional file 1: Fig. S3b).

**Fig. 4:**
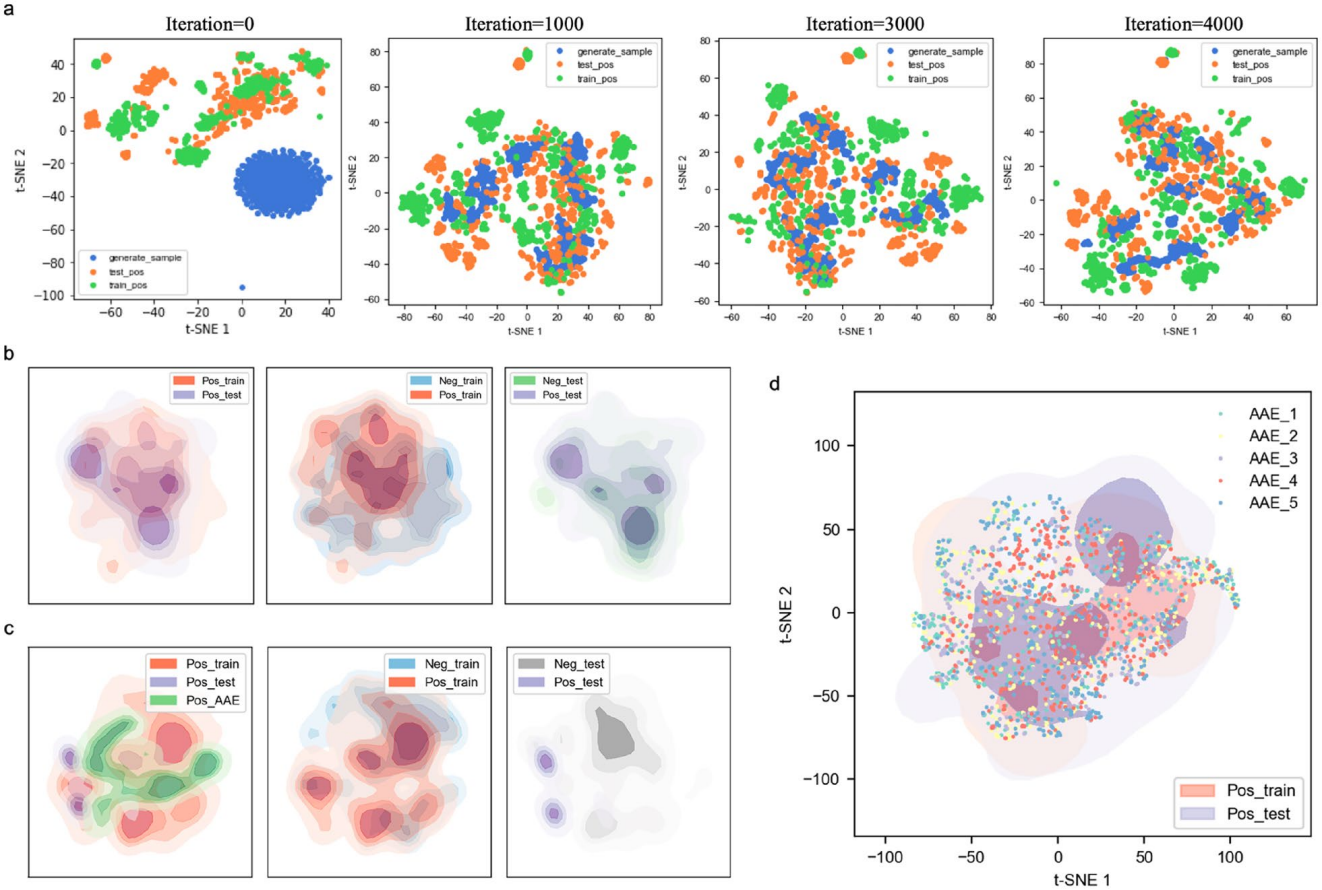
Visualization by t-SNE of the distributions of generated sample, training set and test set. **a** Changes of data distribution during training of PCM-AAE. **b** Distribution of the training data for NB model. **c** Distribution of training set augmented by PCM-AAE. **d** Distribution of generated data from different generators trained with AAE

We tracked the alteration of data space along with training of PCM-AAE. Specifically, the positive training data of PCM-AAE in CV4, positive samples in the CV4 test set, and the generated data were mapped to low dimension every 1000 iterations during the training process (Fig. 4a). Initially, PCM-AAE data space was completely separated from the real dataset, then the “shape” of generated data space became similar to the real data space, and eventually, with the convergence of the model, the generated data points became discretized to cover and expand the real data space gradually.

Subsequently, the data space of PCM-AAE was compared to that of the NB model to investigate the reason why PCM-AAE could reduce the overfitting of NB models. In terms of the NB model (Fig. 4b), the data space of the training set cannot cover that of the test set for both negative data and positive data, explaining why NB models cannot fit the test data well. Such overfitting also occurs more or less in CV1–CV3, which cannot be reduced by tuning the parameters of the models (Additional file 1: Table S1). In addition, it seems difficult for the NB models to distinguish positive samples from negative samples in CV4 test set. This might be ascribed to the imbalanced training set, which resulted in the tendency of NB model to predict data as negative samples. To analyze the PCM-AAE data space, generated samples from the best generator mentioned above (AAE #14) were combined with the dataset of NB model and visualized. As presented in Fig. 4c, although the generated data had relatively less overlap with the real positive data in the presence of negative samples, these novel data points changed the distribution of the data space, yielding more overlap between training set and test set as well as more separated distribution of the positive and negative samples. Encouragingly, the conclusions were consistent when the training set and the test set were randomly regenerated and subjected to the analysis above. The results partially indicated the intuitive idea of PCM-AAE, that is, for the imbalanced dataset, the data distribution can be changed by expanding the data space, so as to improve the generalization ability of the prediction model.

As described above, the 24 generators derived from the PCM-AAE model were used to balance the training set followed by reconstructing the predictors, however, the degrees of improvement on predictors’ performance were varying. We assumed this was possibly owing to the diverse data space generated by different generators. On that account, five generators were randomly selected to generate 500 data points respectively, which were then combined with the real positive data to explore the relationship between different AAE spaces and the real data space (Fig. 4d). As expected, the generated data points overlapped largely with real positive data, and the distribution of data from different AAEs augmented the real data space in a complementary way.

### Training and evaluation of EPA

**Fig. 5:**
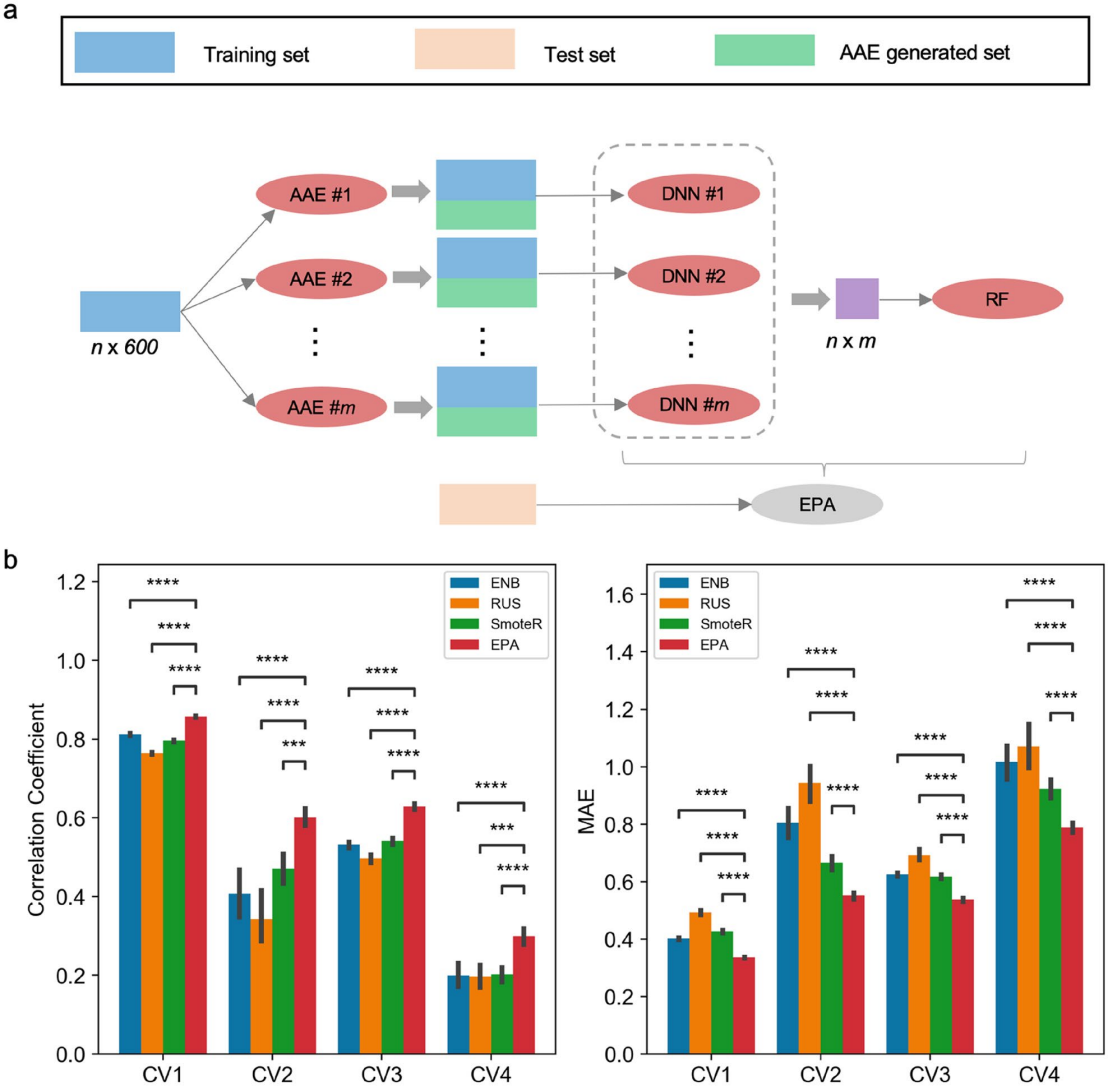
**a** Training process of EPA. The training set (“*n*” and “*m*” represent the number of data points and models respectively) was firstly augmented by *m* AAE models followed by training *m* DNN models. Then DNNs were used to re-predict the training set, generating *n *× *m* data points which were fed into RF model. Finally, the test set was predicted by the trained DNNs and RF model to evaluate the performance of EPA. **b** The performance of EPA compared with ENB, SmoteR and random under sampling (RUS). Statistical significance of the difference between the performance of EPA and ENB was determined by paired t-test. ns: p > 0.05; *p < 0.05; **p < 0.01; ***p < 0.001; ****p < 0.0001

As described above, a single generator might not be enough to complete data space in every case, we proposed an ensemble strategy, termed Ensemble of PCM-AAE (EPA), to enable the different generated spaces to be complemented by each other and thus give a more accurate and robust prediction. The training regime of EPA is shown in Fig. 5a. Firstly, we split the dataset as a training set (*n* data points) and a test set at CV1–CV4 levels respectively. After that, for each generator, the training set was complemented until it balanced, then the oversampled set was fed into the DNN predictors (*n*), which were used to re-predict the original training set. As novel representations of the original training set, the yielded predictions (*n *× *m* dimensions) were then utilized to train the RF model. At the end, the DNN predictors (*m*) coupled with one RF model produced predictions on the corresponding test sets to evaluate the effectiveness of this ensemble strategy. The procedure mentioned above was repeated 20 times for robustness. To be fair, ENB models (ensemble of non-balanced models) with imbalanced training datasets were also trained, evaluated and selected in the same manner as EPA.

Since the essence of PCM-AAE and EPA could be considered as oversampling approaches to solve the imbalanced regression problem, EPA model was not only compared with ENB model and PCM-AAE, but also with classical sampling methods entailing SmoteR and random undersampling (RUS) algorithms. Encouragingly, as shown in Fig. 5b and Additional file 1: Fig. S7, EPA consistently outperformed other methods from CV1 to CV4 levels. As expected, EPA was more robust than PCM-AAE.

In order to rule out the possibility that the superiority of EPA comes from certain similarities of kinase sequences or compounds from training set and test set so that EPA learns the distribution characteristics of representations too easily, we conducted additional studies from CV2 to CV4 to ensure compounds and proteins are strictly “unseen”. In CV2, if any protein sequence from the test set has an identity of more than 25% with any sequence in the training set, it was removed from the test set. In CV3, if any compound from the test set has Tanimoto similarity of more than 80% to any compound in the training set, it was removed from the test set. In CV4, both compounds and sequences in the test set were processed likewise. Subsequently, the performance of ENB and EPA was re-evaluated on the datasets processed as above and the results are shown in Additional file 1: Fig. S8, EPA proved to be significantly better than ENB even under strict conditions.

To further examine the superiority of EPA model over ENB, assessments were conducted on five external independent datasets. It is noteworthy that the data points presented in the external sets were removed from the original training set (before the split in cross-validation folds) when constructing validation datasets. Apart from assessing whether EPA fits the set better as a regression model via PCC, MAE and MSE, we also employed metrics of AUC (area under ROC), F1 score, precision and recall with the cut-off of 6 (corresponding to the concentration of 1 µM) to evaluate the ability of EPA to deal with classification problems. The results are summarized in Table 2. Overall, the performance of EPA was more or less more favorable than ENB. Interestingly, in terms of PKIS1 dataset, neither ENB nor EPA could achieve enough accuracy. We supposed this might be owing to data inconsistency among datasets that derived from different biochemical assays. To verify this, the PCCs between the shared data points from every two datasets were calculated (Additional file 1: Fig. S9). As expected, PKIS1 dataset did show a poor consistency to the training set with a PCC of 0.52, while other datasets that are more relevant to the training set yielded better predictions. Additionally, we found the increased classification ability of EPA compared with ENB seemed to arise from the improvement of recall score, indicating that the capability of EPA to capture positive samples is stronger than ENB

**Table 2:** Summary of ENB and EPA performance on external datasets

Dataset	Method	Regression metrics			Classification metrics			
		PCC	MAE	MSE	AUC	F1 score	Precision	Recall
Metz	ENB	0.47	0.51	0.49	0.81	0.45	0.39	0.54
	EPA	**0.54**	**0.44**	**0.39**	**0.84**	**0.49**	**0.41**	**0.61**
Davis	ENB	0.38	1.37	0.90	0.67	0.58	**0.77**	0.46
	EPA	**0.42**	**1.10**	**0.79**	**0.69**	**0.64**	0.76	**0.55**
PKIS1	ENB	0.29	0.64	0.59	0.75	0.25	0.21	0.30
	EPA	**0.33**	**0.56**	**0.44**	**0.79**	**0.26**	**0.22**	**0.32**
KinaseSafari	ENB	0.33	1.1	2.14	0.68	0.56	0.74	0.45
	EPA	**0.44**	**1.01**	**1.72**	**0.73**	**0.61**	**0.77**	**0.51**
MRC	ENB	0.38	0.83	1.14	0.68	0.33	0.53	0.24
	EPA	**0.45**	**0.78**	**0.99**	**0.74**	**0.38**	**0.54**	**0.30**

The bold number denotes the better result between ENB and EPA for predicting the corresponding external dataset

### Applicability of EPA on kinase profile prediction and virtual screening

**Fig. 6:**
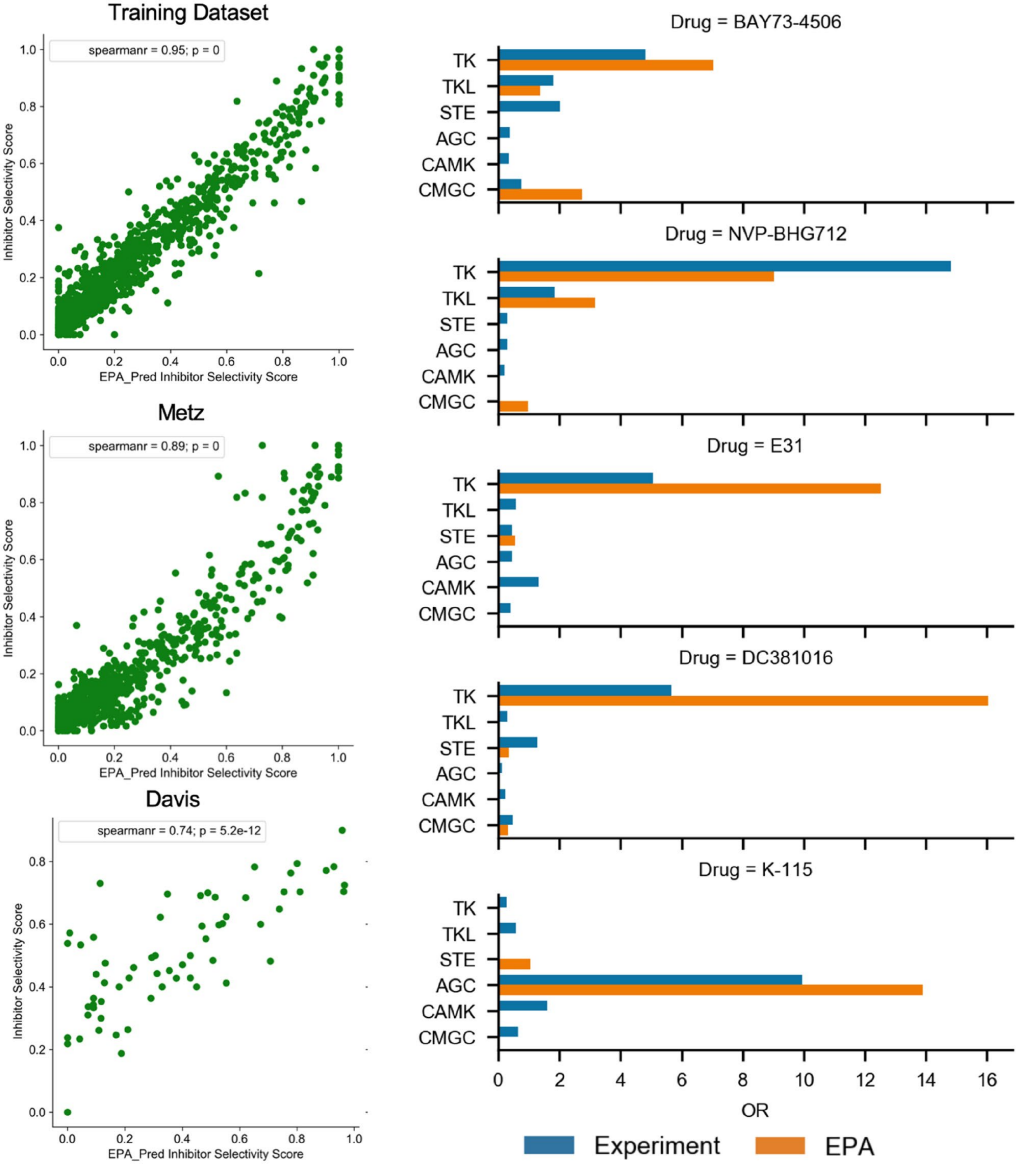
**a** Scatterplots of EPA-predicted selectivity score and experimental selectivity score of inhibitors in various sets. **b** Comparison of experimental odd ratio (OR) and EPA-predicted OR for five drugs

Detecting off-target effects or the kinase profiling of kinase-specific inhibitors is vital for portraying the attribution of drugs. To quantify the model’s capability to predict off-target effects of compounds, the selectivity score was defined as the ratio of the number of hits to that of all tested kinases [44]. Intuitively, for a given inhibitor, a lower selectivity score indicates better selectivity. Hence, we re-predicted the training set by EPA, generating predicted selectivity of each compound for correlation analysis with the compound selectivity measured by experiments. Another two sets, Davis’s and Metz’s, with relatively complete kinase panels for different compounds, were also analyzed in the same way. As presented in Fig. 6a, experimental selectivity scores from the 3 datasets correlate closely with corresponding predictions by the EPA model, with Spearman correlation coefficient of 0.95, 0.89, and 0.74 respectively, which again outperformed ENB (Additional file 1: Fig. S10). To further validate whether EPA could accurately predict subfamily selectivity, Li’s dataset that contains the kinase profile of five inhibitors absent from the datasets we employed so far was analyzed. Meanwhile, OR (odds ratio) [45] was also introduced to quantify subfamily selectivity. The major targeted subfamilies of the compounds predicted by EPA accord well with experimental results (Fig. 6b).

**Fig. 7:**
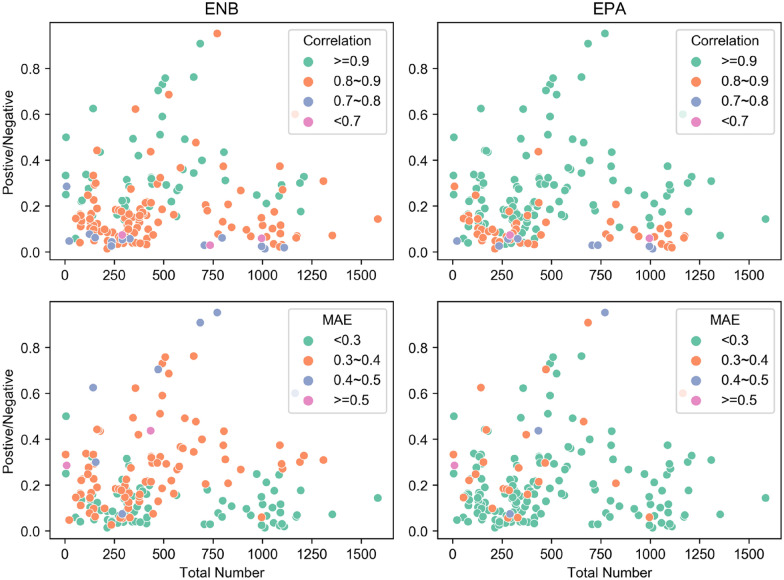
The ratio of positive samples to negative samples for each kinase plotted against total number of data points in training set, based on the predictions by ENB and EPA

Another important approach to drug discovery is structure-based virtual screening. Given the desirable performance of EPA in CV3, we examined its effectiveness to recall active compounds for a given kinase and the potential of becoming an efficient alternative to conventional structure-based virtual screening methods. Firstly, we analyzed the training set grouped by targets. In each subset, PCC and MAE were used to assess the predictive power of the regressor. Theoretically, EPA can address the machine learning problem even when training data were insufficient. Thus, we next analyzed the relationship between model performance and data composition including the number of data points and degree of data imbalance. As presented in Fig. 7, on the same training set, the number of well-fitted kinases by EPA was far more than that of ENB, especially for kinases with few or imbalanced data points.

**Fig. 8:**
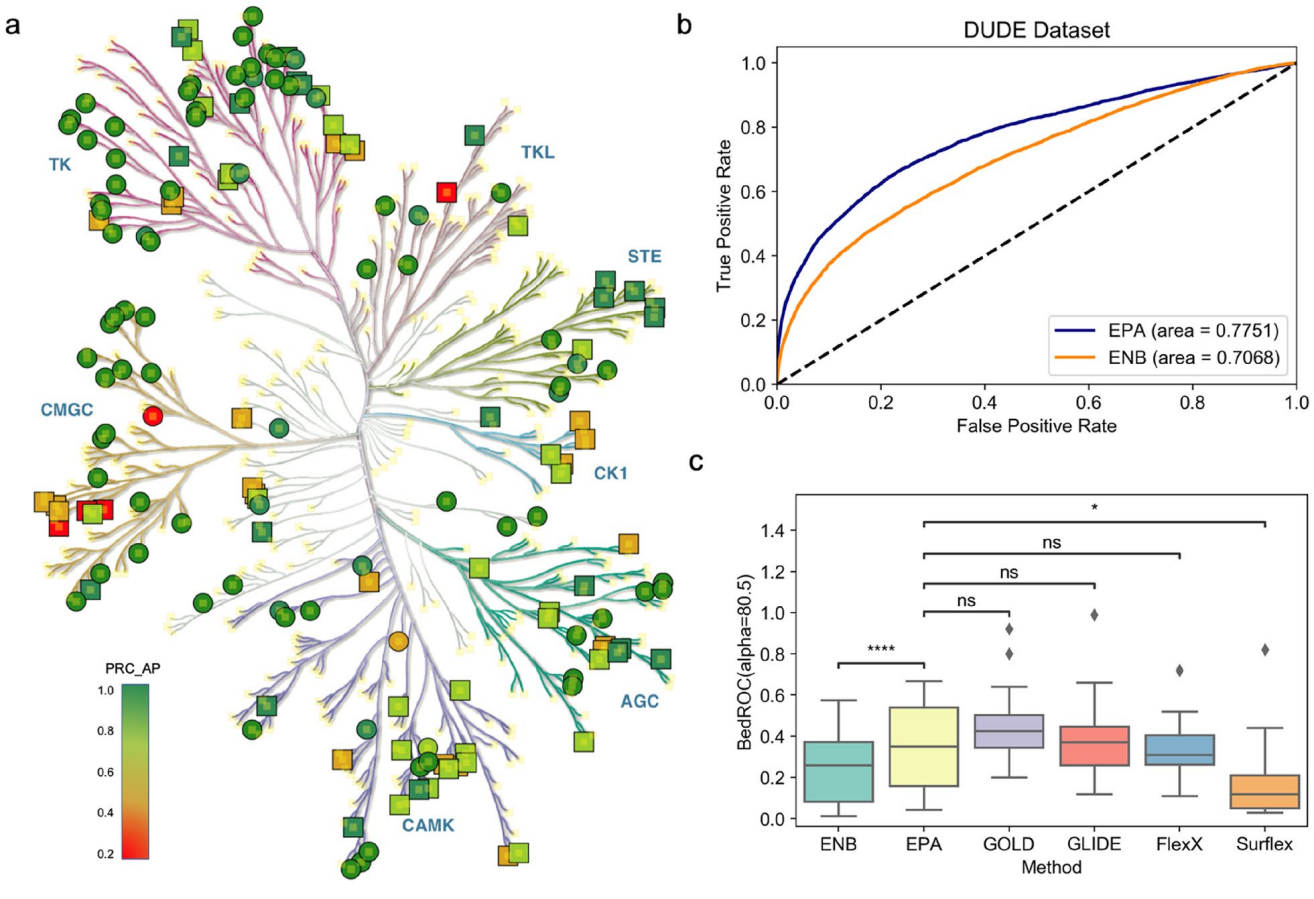
**a** Phylogenetic tree to display the performance of EPA on kinases from the Metz’s set. Circles represent the kinases included in training set. Squares represent the kinases absent from training set. **b** ROC analysis of EPA and ENB predictions to DUD-E dataset. **c** Distribution of BEDROC (alpha = 80.5) covering 26 kinase targets in DUD-E for EPA and docking programs. Statistical significance of the difference between the performance of EPA and other methods was determined with paired t-test. ns: p > 0.05; *p < 0.05; **p < 0.01; ***p < 0.001; ****p < 0.0001

Next, we delved into the kinases in Metz’s dataset to further investigate the capability of ENB and EPA to screen active compounds (Fig. 8a and Additional file 1: Fig. S11). The set was divided into two subsets, where one subset included the kinases presented in the training set (presented as circles) and the kinases in the other subset were absent from the training set (presented as squares). Although both ENB and EPA generated considerable predictions to most kinases that had been seen by the model, EPA topped ENB on most kinases that were previously unseen by the models, further suggesting the effectiveness of our strategy. Encouraged by these results, we evaluated EPA with 26 kinases in the Directory of Useful Decoys-Enhanced (DUD-E) database [46] to see if EPA discriminates true binders from decoy molecules. As shown in Fig. 8b, EPA achieved superior predictions over ENB again with an average AUC of 0.77, which is quite high for a non-docking-based predictive method so far to the best of our knowledge. In addition, the performance of EPA was comparable with classical docking algorithms including GOLD, Glide, SurFlex, and FlexX in terms of BEDROC scores at alpha of 80.5 based on the benchmark work of Chaput et al. [47] (Fig. 8c).

Overall, EPA shows superior performance compared with ENB, yet it also has its limitations. Firstly, like most CPI models, EPA is unable to address the challenge of activity cliff and thus cannot be applied to compound structure optimization, which may be overcome by more chemically- and biologically-relevant representations of compounds or proteins. Secondly, frequent mode collapse of GANs [48] probably makes the data points generated by AAE not diversified enough to achieve effective data augmentation, which has been partially resolved by our ensemble strategy, and novel GAN framework such as WGAN, VEEGAN and VirtualGAN [49–51] may contribute to additional improvement. Thirdly, the EPA model relies on the ensemble of multiple PCM-AAE models, making it not convenient enough to train and use under a limited computational budget, a lighter yet promising network may be obtained via ensemble knowledge distillation or model compression in future work [52] .

## Conclusions

To conclude, we devised EPA, a framework against imbalanced regression problem and applied it to improving the predictive power of proteochemometrics (PCM) model which focused on kinase-compound binding affinity prediction. We trained a number of AAE models (PCM-AAE) using active compounds to generate diverse feature space then made an ensemble to augment and balance global data space. This strategy not only outperformed classical non-balanced PCM models at rigorous fourth cross-validation level, but also showed better and more robust performance than classical methods against imbalanced regression tasks. We envision that EPA is a proof-of-concept implementation of a generative model in exploring PCM data space, which is expected to be applicable in various drug discovery scenarios where abundant training data are not available.

## Supplementary Information

**Additional file 1: Figure S1.** **a** Datarepresentation. Combination 1: concatenating 100-dimensional compoundembeddings with the sum of three 100-dimensional 3-g protein sequence embeddings. Combination 2: concatenating300-dimensional compound embeddings with the sum of three 300-dimensional 3-g proteinsequence embeddings. Combination 3: concatenating 300-dimensional compound embeddings with the concatenation ofthree 100-dimensional 3-g protein sequence embeddings. **b** Performancecomparison of models fedwith different data representations. **FigureS2.** **a** Partial architecture ofPCM-GAN. The input of generator issampled from low-dimensional Gaussian distribution (orange).The output of generator is 600-dimensional feature (purple) + one-dimensionalcorresponding label/activity (green). **b** Partial architecture of PCM-AAE. The input of encoder and output of decoder are 600-dimensionalfeature (purple) + one-dimensional corresponding label/activity (green). **FigureS3.** **a** Cumulative variance explained by thenumber of components in PCA. **b** Runningtime behavior of t-SNE and PCA+t-SNE methods for dimensionality reduction. **Figure S4.** Performance of fourdifferent machine learning models at four levels. (CV2: new target prediction; CV3:new drug prediction; CV4: pair prediction of new target and new drug). **Figure S5.** Lossplot of PCM-GAN which **a** didnot use batch-normalization (BN); **b**used BN in the generator. **FigureS6.** Performancecomparison among NB model (non-balanced model) and 24 reconstructed models fedwith data augmented by 24 generators respectively. **Figure S7.** Performance comparison between PCM-AAEand EPA. Statistical significance of the difference between theperformance of EPA and PCM-AAE was determined by paired t-test. ns: p > 0.05;*: p < 0.05; **: p < 0.01; ***: p < 0.001; ****: p < 0.0001. **Figure S8.** Performance comparison betweenENB and EPA on stricter “unseen” test sets. Statistical significance of thedifference between the performance of EPA and ENB was determined by pairedt-test. ns: p > 0.05; *: p < 0.05; **: p < 0.01; ***: p < 0.001;****: p < 0.0001. **FigureS9.** Correlationcoefficient between every two datasets. **FigureS10.** Scatterplots of ENB predicted selectivity score and experimentalselectivity score of inhibitors in various sets. **Figure S11.** Phylogenetic tree to display the performance of ENB onkinases from the Metz’s set. Circles represent the kinases included in trainingset. Squares represent the kinases excluded from training set. **Table S1.** Non-balanced model performancein training set and test set. **Algorithm S1.** TrainingPCM-AAE.

## Data Availability

The code for EPA is available at http://github.com/xybai-dev/EPA. Dataset of Kinase SARfari was downloaded from http://ftp.ebi.ac.uk/pub/databases/chembl/. MRC dataset was downloaded from https://www.ppu.mrc.ac.uk/. The other data used in this paper are supported by the corresponding reference.

## Abbreviations

AAE: Adversarial auto-encoder
AP: Average precision
AUC: Area under the ROC curve
DL: Deep learning
DNN: Deep neural network
CPI: Compound–protein interaction
ECFP: Extended-connectivity circular fingerprints
GAN: Generative adversarial network
MSE: Mean squared error
MAE: Mean absolute error
PCA: Principal component analysis
PCM: Proteochemometrics
PDB: Protein data bank
POC: Percent of control
PRC: Precision recall curve
ReLU: Rectified linear unit
RF: Random forest
ROC: Receiver operating characteristic
*t*-SNE: t-Distributed Stochastic Embedding
