# Supplementary material for: Exploration and augmentation of pharmacological space via adversarial auto-encoder model for facilitating kinase-centric drug development
Source: J Cheminform. 2021 Dec 6;13:95. doi: 10.1186/s13321-021-00574-4 (PMC8650415; doi:10.1186/s13321-021-00574-4)
Supplement: Supplementary file 1 — Additional file 1: Figure S1. a Datarepresentation. Combination 1: concatenating 100-dimensional compoundembeddings with the sum of three 100-dimensional 3-g protein sequence embeddings. Combination 2: concatenating300-dimensional compound embeddings with the sum of three 300-dimensional 3-g proteinsequence embeddings. Combination 3: concatenating 300-dimensional compound embeddings with the concatenation ofthree 100-dimensional 3-g protein sequence embeddings. b Performancecomparison of models fedwith different data representations. FigureS2. a Partial architecture ofPCM-GAN. The input of generator issampled from low-dimensional Gaussian distribution (orange).The output of generator is 600-dimensional feature (purple) + one-dimensionalcorresponding label/activity (green). b Partial architecture of PCM-AAE. The input of encoder and output of decoder are 600-dimensionalfeature (purple) + one-dimensional corresponding label/activity (green). FigureS3. a Cumulative variance explained by thenumber of components in PCA. b Runningtime behavior of t-SNE and PCA+t-SNE methods for dimensionality reduction. Figure S4. Performance of fourdifferent machine learning models at four levels. (CV2: new target prediction; CV3:new drug prediction; CV4: pair prediction of new target and new drug). Figure S5. Lossplot of PCM-GAN which a didnot use batch-normalization (BN); bused BN in the generator. FigureS6. Performancecomparison among NB model (non-balanced model) and 24 reconstructed models fedwith data augmented by 24 generators respectively. Figure S7. Performance comparison between PCM-AAEand EPA. Statistical significance of the difference between theperformance of EPA and PCM-AAE was determined by paired t-test. ns: p > 0.05;*: p < 0.05; **: p < 0.01; ***: p < 0.001; ****: p < 0.0001. Figure S8. Performance comparison betweenENB and EPA on stricter “unseen” test sets. Statistical significance of thedifference between the performance of EPA and ENB was determined by pairedt-test. [file 13321_2021_574_MOESM1_ESM.docx]

**Additional material**

**Exploration and Augmentation of Pharmacological Space via Adversarial Autoencoder Model for Facilitating Kinase-centric Drug Development**

Xinyu Bai^1^, Yuxin Yin^2,*^

^1^Department of Pathology, School of Basic Medical Sciences, Peking University Health Science Center, Beijing 100191, China,

^2^Institute of Systems Biomedicine, School of Basic Medical Scienc-es, Peking University Health Science Center, Beijing 100191, China; Peking-Tsinghua Center for Life Sciences, Peking University Health Science Center, Beijing 100191, China.

*Correspondence:

Yuxin Yin, M.D., Ph.D.

Institute of Systems Biomedicine

School of Basic Medical Sciences

Peking University Health Science Center

Beijing 100191, P.R. China

Phone: (86) 10-8280-1237

Fax: (86) 10-8280-1380

E-mail: [yinyuxin@hsc.pku.edu.cn](mailto:yinyuxin@hsc.pku.edu.cn)


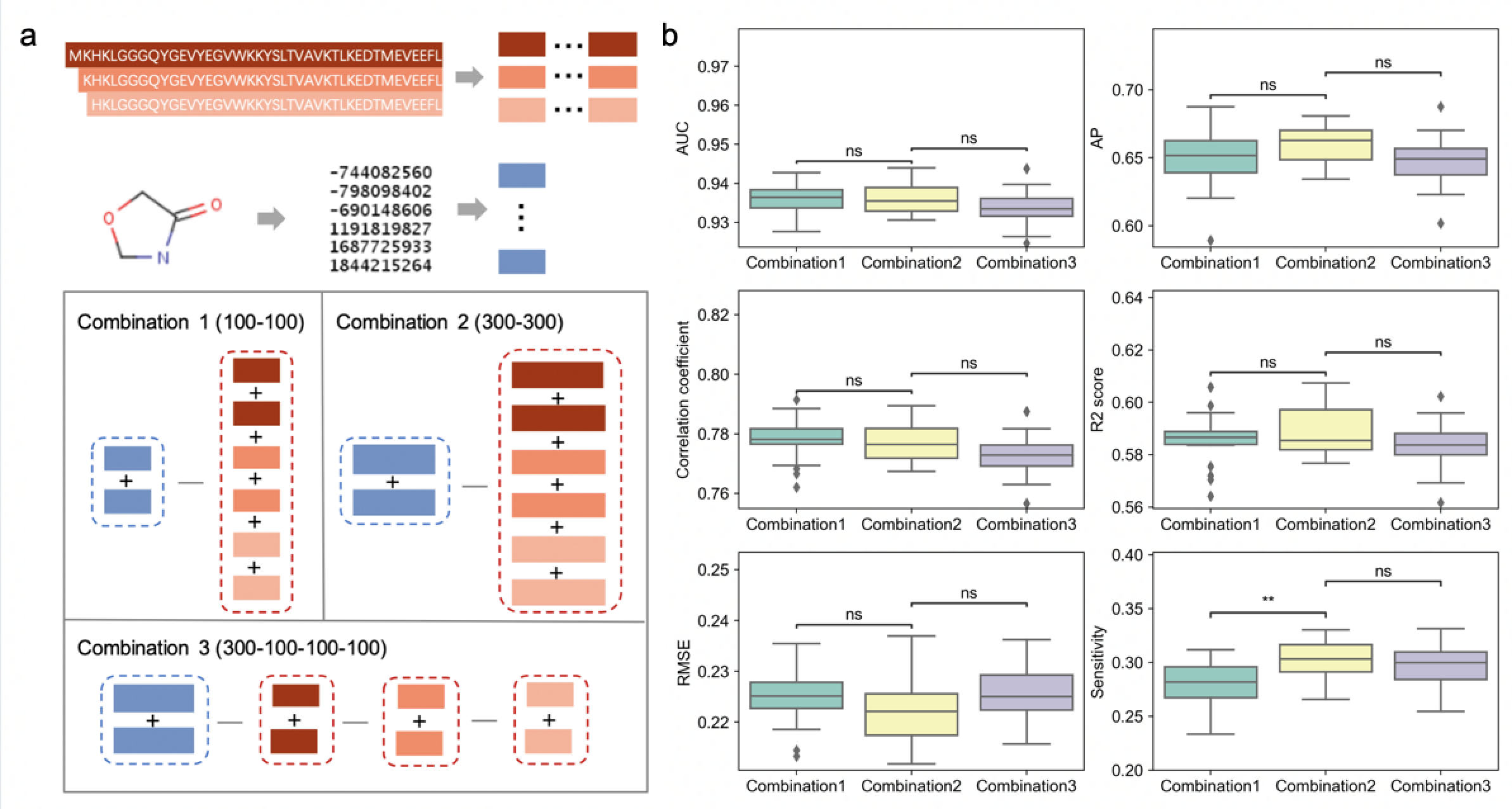


**Fig. S1 a** Data representation. Combination 1: concatenating 100-dimensional compound embeddings with the sum of three 100-dimensional 3-gram protein sequence embeddings. Combination 2: concatenating 300-dimensional compound embeddings with the sum of three 300-dimensional 3-gram protein sequence embeddings. Combination 3: concatenating 300-dimensional compound embeddings with the concatenation of three 100-dimensional 3-gram protein sequence embeddings. **b** Performance comparison of models fed with different data representations.


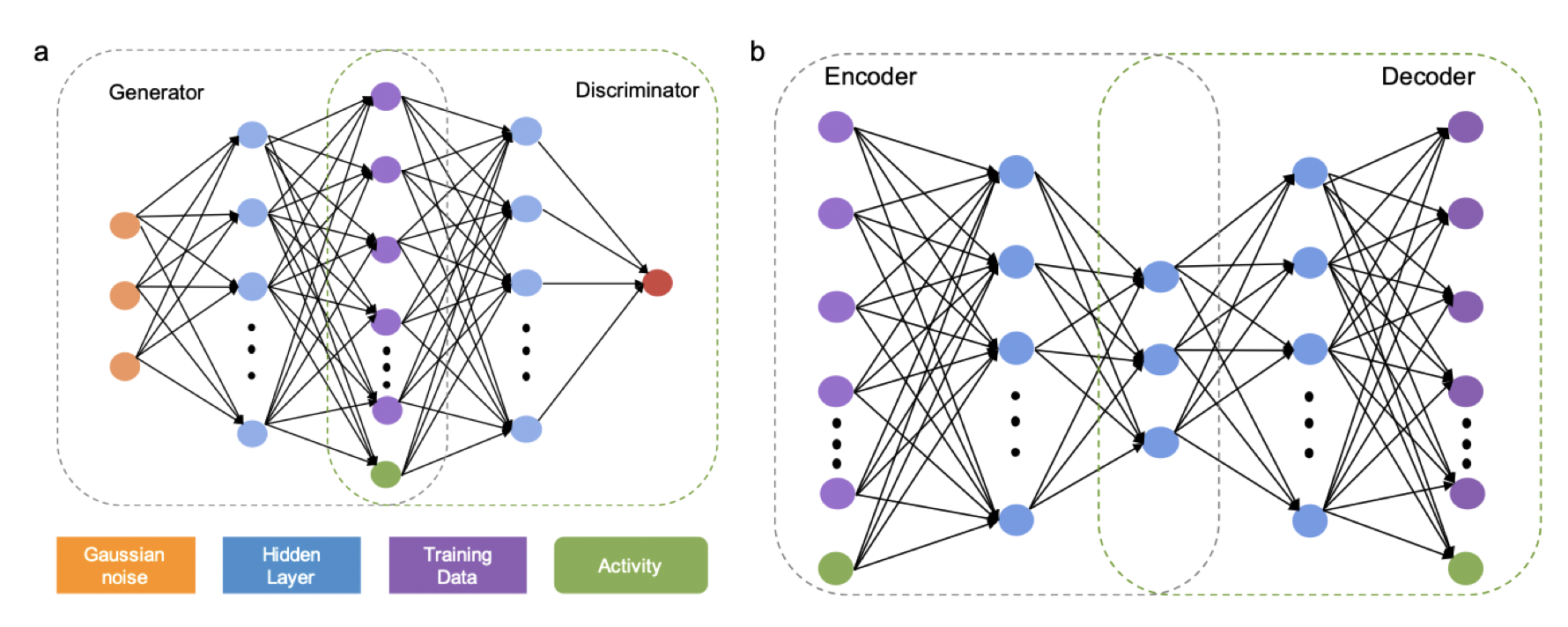


**Fig. S2 a** Partial architecture of PCM-GAN. The input of generator is sampled from low- dimensional Gaussian distribution (orange). The output of generator is 600-dimensional feature (purple) + one-dimensional corresponding label/activity (green). **b** Partial architecture of PCM-AAE. The input of encoder and output of decoder are 600-dimensional feature (purple) + one-dimensional corresponding label/activity (green).


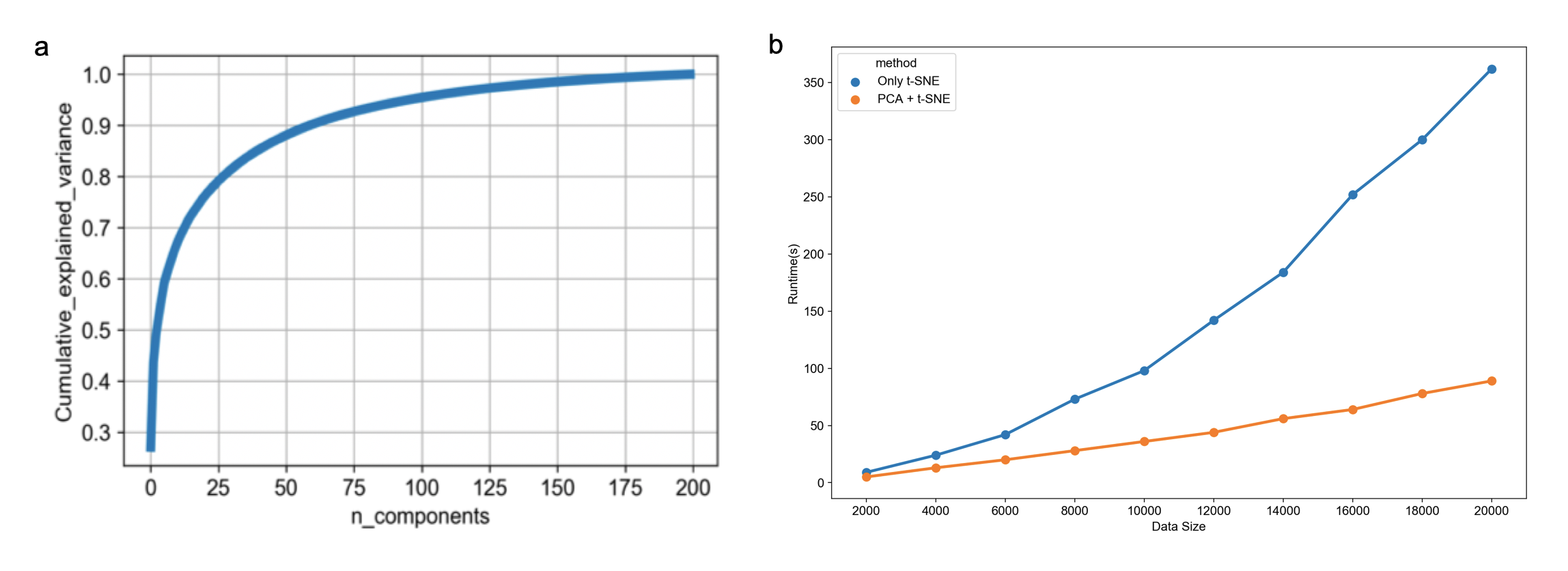


**Fig. S3 a** Cumulative variance explained by the number of components in PCA. **b** Running time behavior of t-SNE and PCA+t-SNE methods for dimensionality reduction.


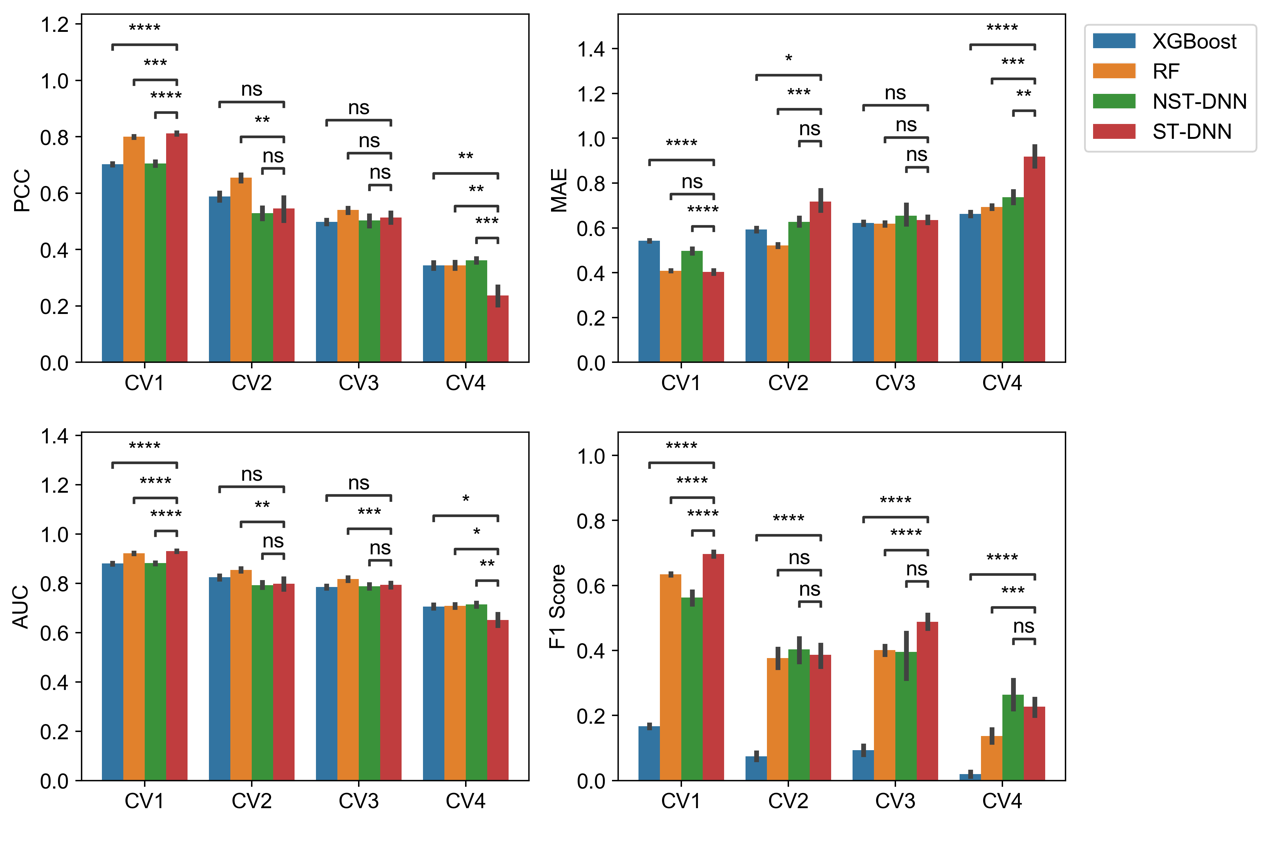


**Fig. S4** Performance of four different machine learning models at four levels. (CV2: new target prediction; CV3: new drug prediction; CV4: pair prediction of new target and new drug).


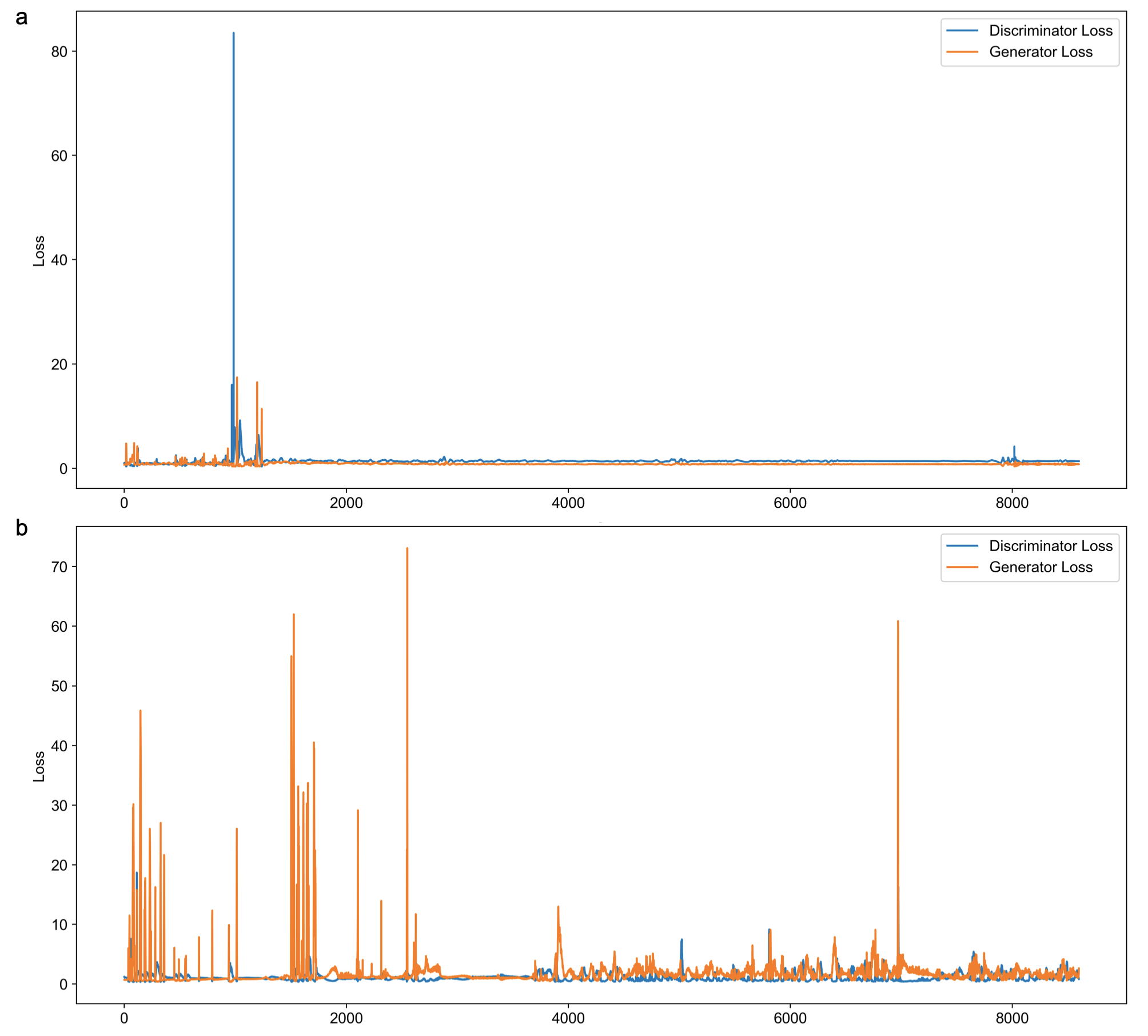


**Fig. S5** Loss plot of PCM-GAN which **a** did not use batch-normalization (BN); **b** used BN in the generator.


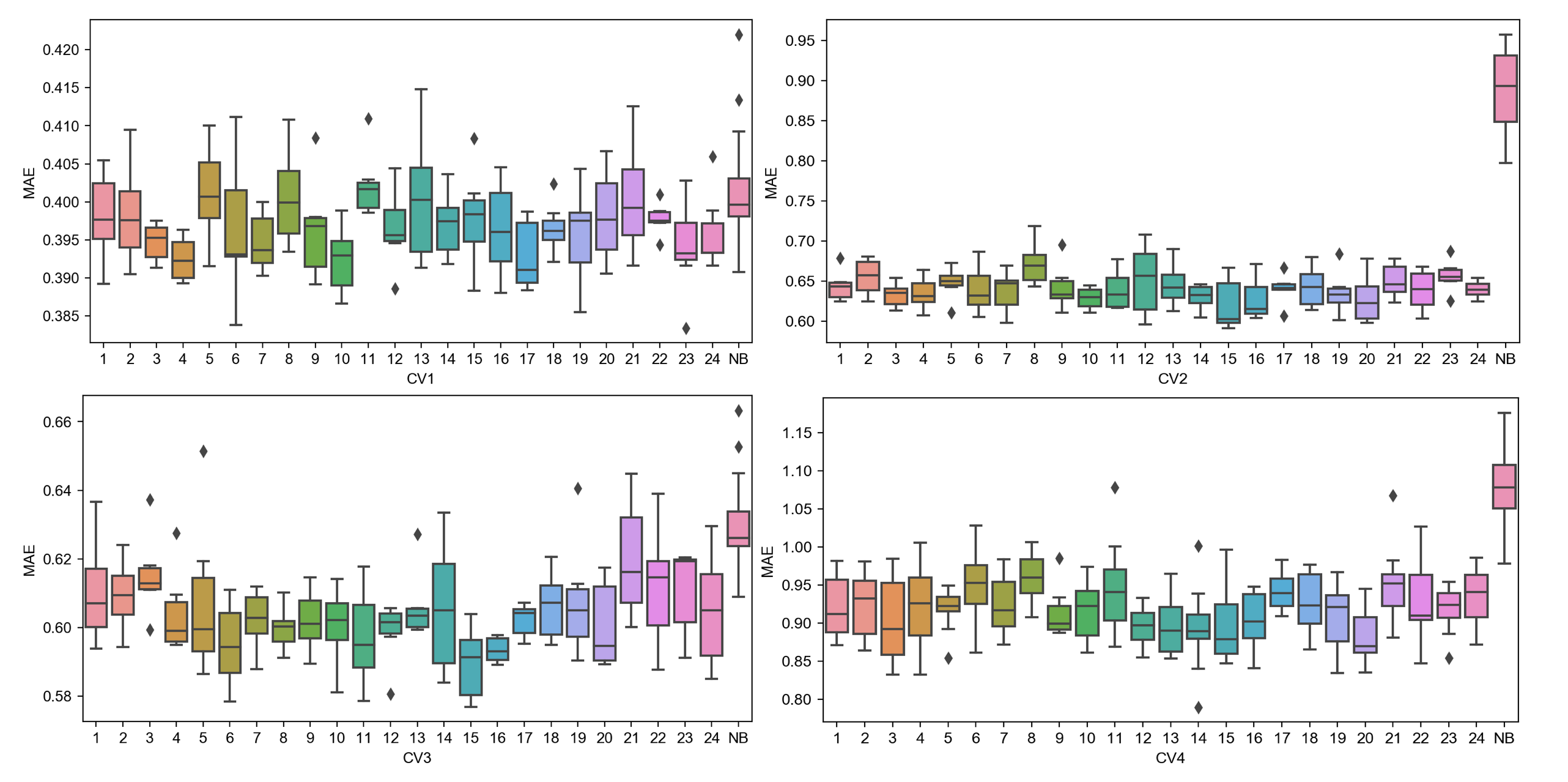


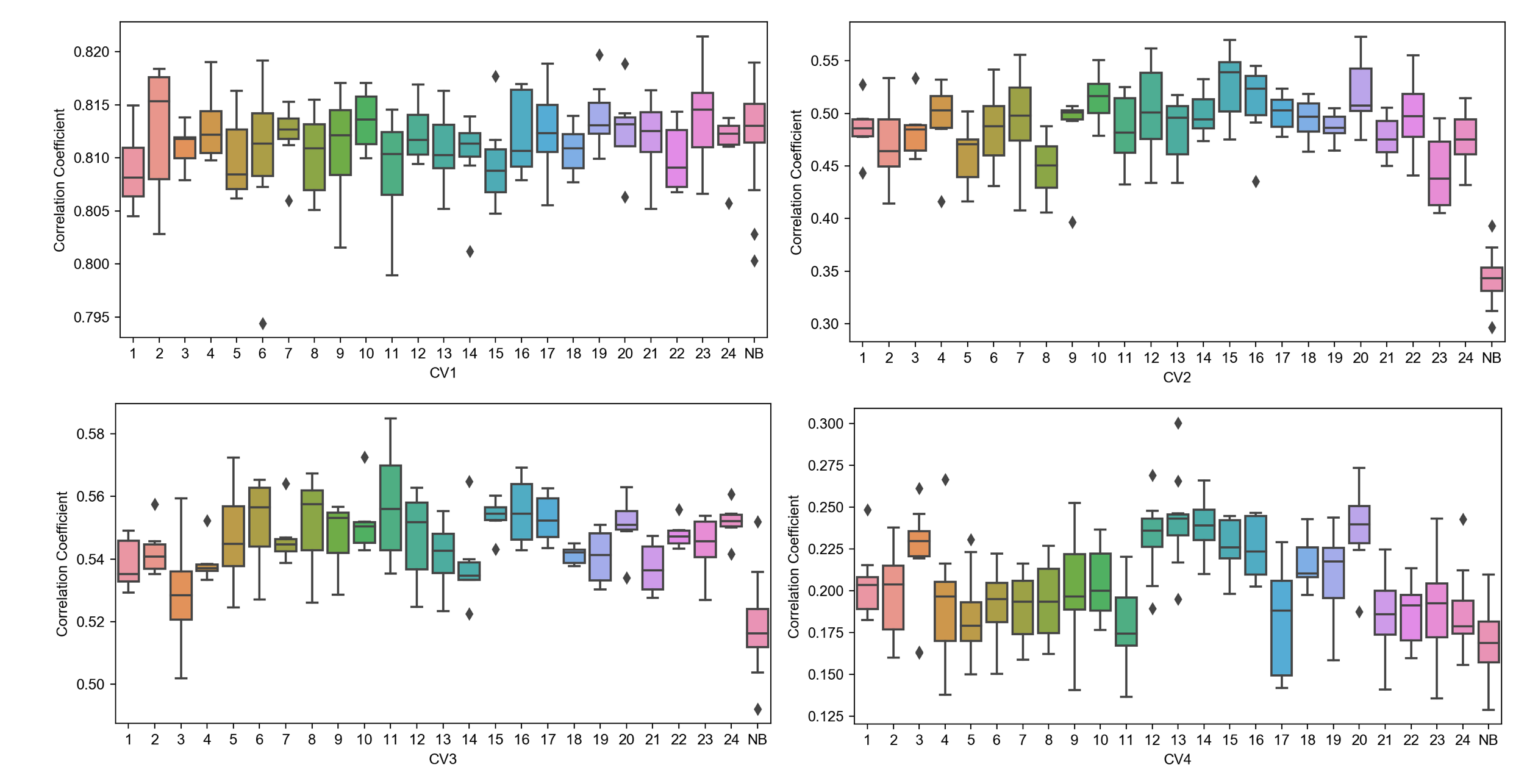


**Fig. S6** Performance comparison among NB model (Non-Balanced model) and 24 reconstructed models fed with data augmented by 24 generators respectively.


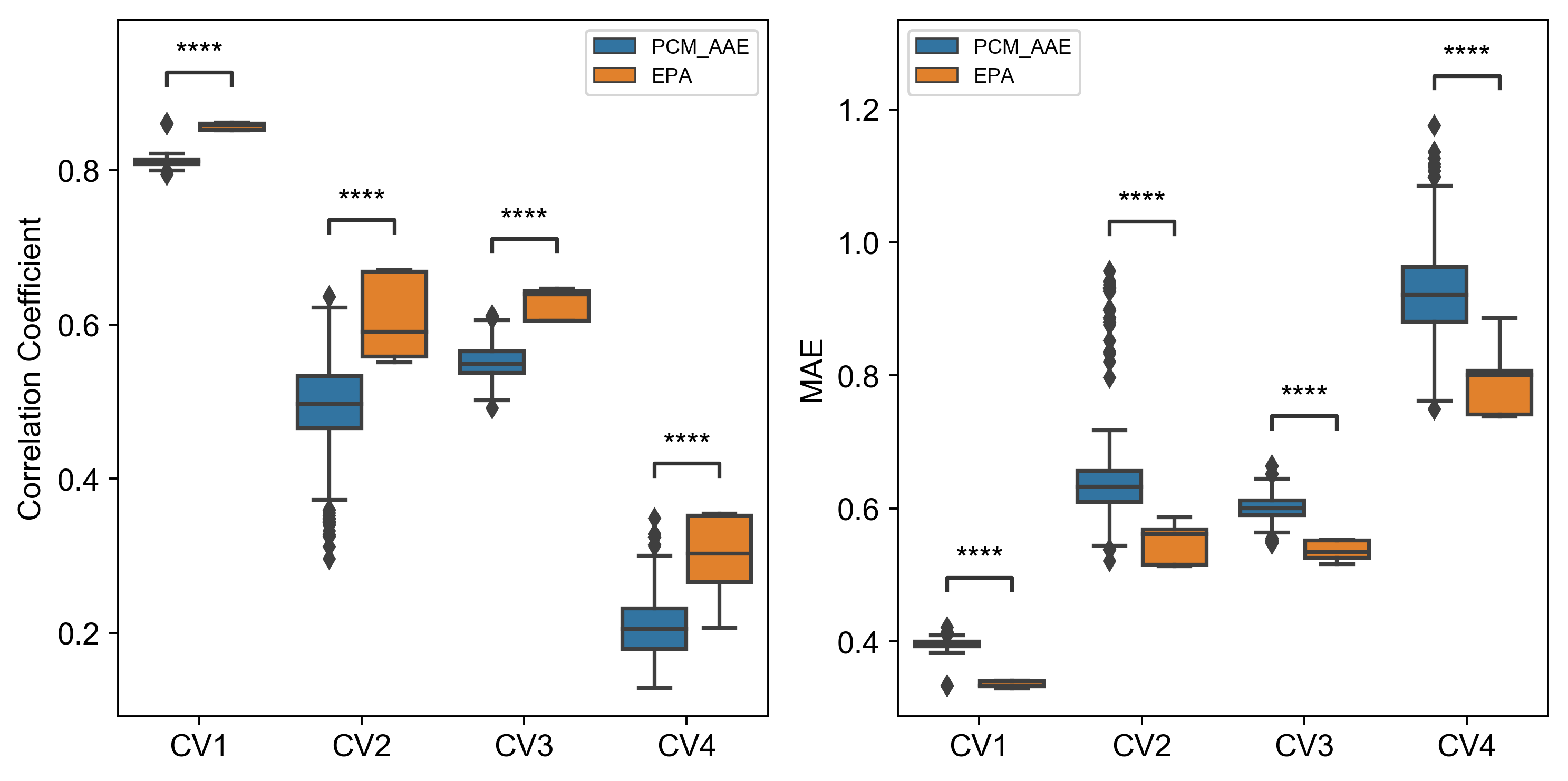


**Fig. S7** Performance comparison between PCM-AAE and EPA. Statistical significance of the difference between the performance of EPA and PCM-AAE was determined by paired t-test. ns: p>0.05; *: p < 0.05; **: p < 0.01; ***: p < 0.001; ****: p<0.0001.


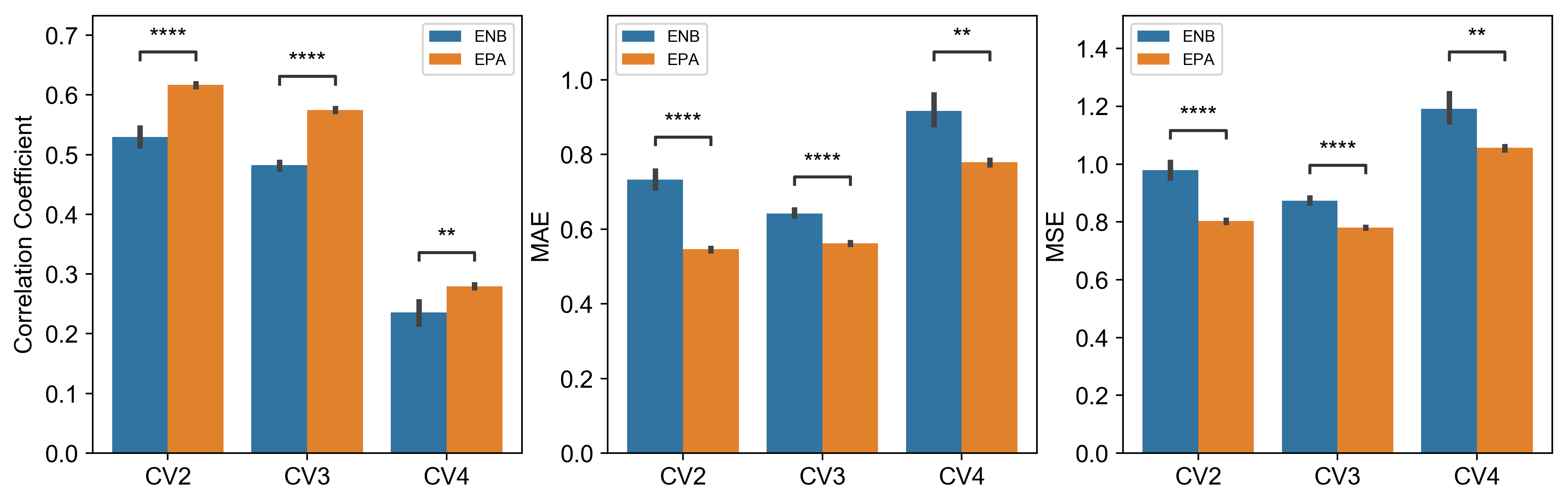


**Fig. S8**. Performance comparison between ENB and EPA on stricter “unseen” test sets. Statistical significance of the difference between the performance of EPA and ENB was determined by paired t-test. ns: p>0.05; *: p < 0.05; **: p < 0.01; ***: p < 0.001; ****: p<0.0001.


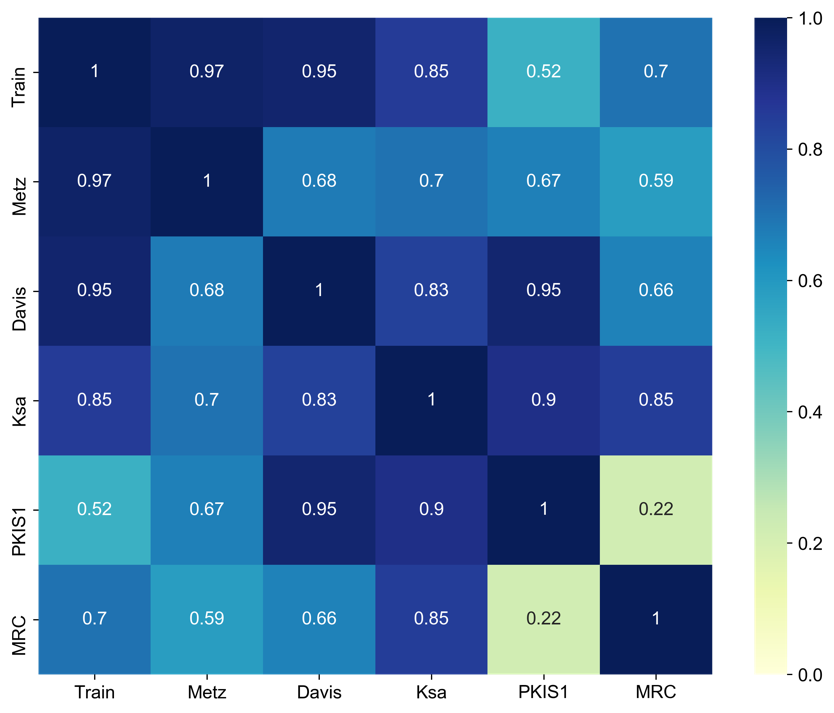


**Fig. S9** Correlation coefficient between every two datasets.


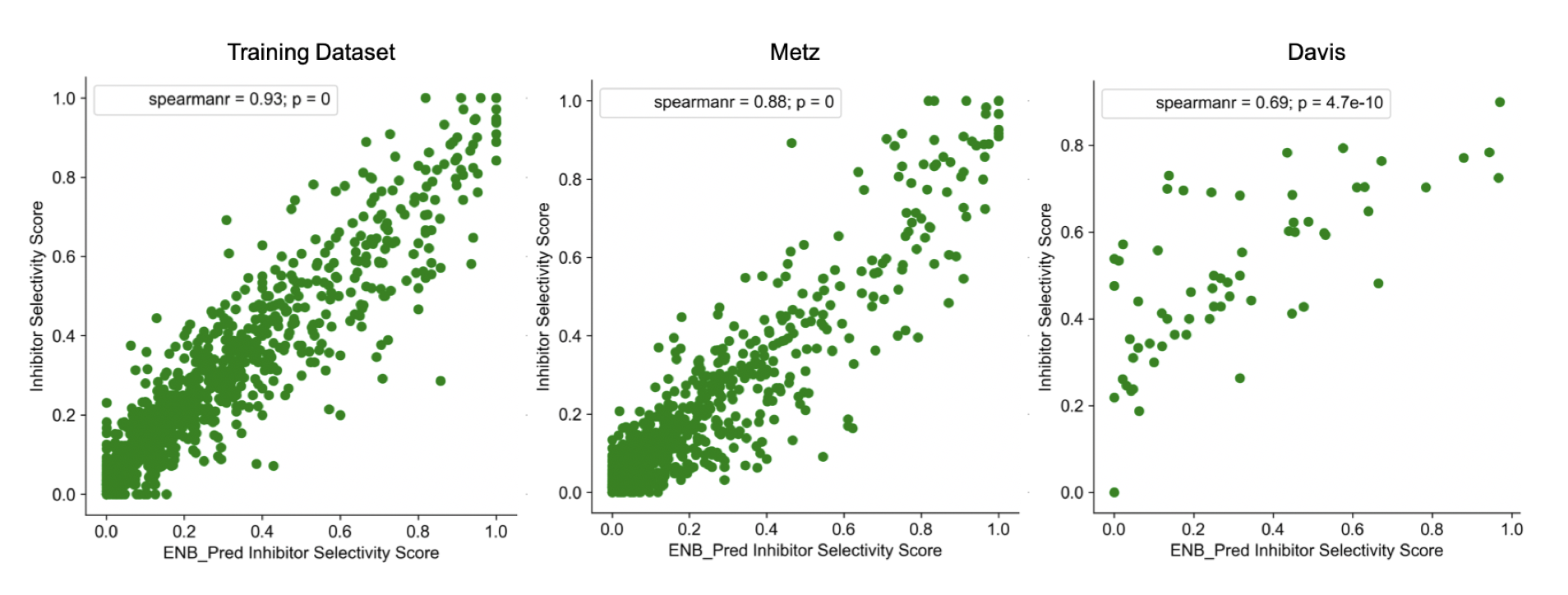


**Fig. S10** Scatterplots of ENB predicted selectivity score and experimental selectivity score of inhibitors in various sets.


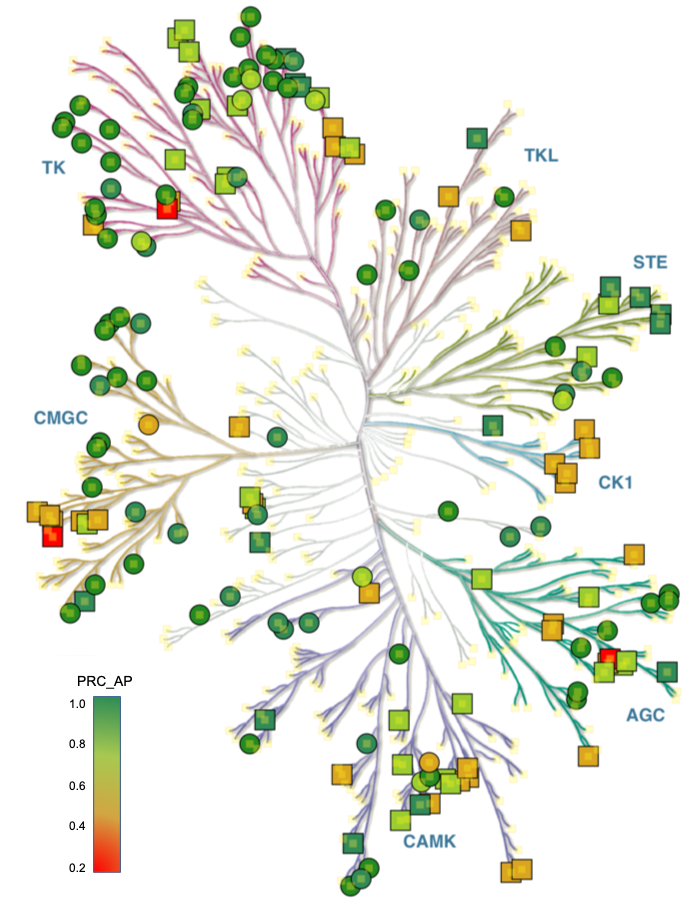


**Fig. S11** Phylogenetic tree to display the performance of ENB on kinases from the Metz’s set. Circles represent the kinases included in training set. Squares represent the kinases excluded from training set.

**Table S1.** Non-balanced model performance in training set and test set.

| CV | Dataset | PCC | MAE | MSE | AUC | F1 score |
| --- | --- | --- | --- | --- | --- | --- |
| CV1 | Train | 0.91±0.003 | 0.27±0.005 | 0.15±0.005 | 0.97±0.002 | 0.80±0.009 |
|  | Test | 0.81±0.004 | 0.4±0.011 | 0.32±0.015 | 0.93±0.003 | 0.7±0.012 |
| CV2 | Train | 0.93±0.008 | 0.28±0.009 | 0.14±0.004 | 0.98±0.000 | 0.84±0.006 |
|  | Test | 0.55±0.053 | 0.72±0.049 | 0.98±0.149 | 0.8±0.025 | 0.39±0.033 |
| CV3 | Train | 0.94±0.003 | 0.24±0.005 | 0.12±0.007 | 0.98±0.001 | 0.84±0.009 |
|  | Test | 0.51±0.028 | 0.63±0.022 | 0.78±0.055 | 0.79±0.015 | 0.49±0.036 |
| CV4 | Train | 0.95±0.004 | 0.21±0.009 | 0.08±0.002 | 0.98±0.003 | 0.88±0.004 |
|  | Test | 0.24±0.054 | 0.92±0.077 | 1.57±0.252 | 0.65±0.041 | 0.23±0.042 |

| **Algorithm S1:** Training PCM-AAE |
| --- |
| **Input**: The positive set of CV4 training data (TrainData), batch size and the learning rate.  **Output:** Parameters of generator and decoder.  **Procedure:**  **for** k=1 to num_epoch **do**:   1. Draw a batch of positive set and negative set respectively based on batch size (64). At the same time, sample the 64-dimensional noise from Gaussian distribution. 2. Minimize the reconstruction loss of auto-encoder. 3. Match the generated data to positive data distribution by training generator and discriminator.   **If** length(6 < TrainData[-1] < 11)/length(TrainData) >0.9:  Save generator and decoder.  **end** |
